# Following The Trail: Factors Underlying the Sudden Expansion of the Egyptian Mongoose (*Herpestes ichneumon*) in Portugal

**DOI:** 10.1371/journal.pone.0133768

**Published:** 2015-08-12

**Authors:** Tânia Barros, João Carvalho, Maria João Ramos Pereira, Joaquim P. Ferreira, Carlos Fonseca

**Affiliations:** 1 Departamento de Biologia & CESAM (Centro de Estudos do Ambiente e do Mar), Universidade de Aveiro, Campus Universitário Santiago 3810–193 Aveiro, Portugal; 2 Servei d' Ecopatologia de Fauna Salvatge (SEFaS), Departament de Medicina i Cirurgia Animals, Universitat Autonoma de Barcelona (UAB), 08193 Bellaterra, Barcelona, Spain; 3 PPGBAN, Departamento de Zoologia, Instituto de Biociências, Universidade Federal do Rio Grande do Sul, Av. Bento Gonçalves 9500, Porto Alegre RS 91540–000, Brasil; University of California Davis, UNITED STATES

## Abstract

Species range-limits are influenced by a combination of several factors. In our study we aimed to unveil the drivers underlying the expansion of the Egyptian mongoose in Portugal, a carnivore that was confined to southern Portugal and largely increased its range during the last three decades. We evaluated the expansion of the species in three periods (1980-1990, 1990-2000 and 2000-2010), by projecting the presence/absence data of the species in each temporal range and proposed four hypotheses to explain this sudden expansion associated to changes in the barrier effects of human infrastructure and topographic features, and in the availability of suitable areas due to climate change or land use. An exploratory analysis was made using Spearman rank correlation, followed by a hierarchical partitioning analysis to select uncorrelated potential explanatory variables associated with the different hypotheses. We then ran Generalized Linear Models (GLM) for every period for each hypothesis and for every combination of hypotheses. Our main findings suggest that dynamic transitions of land-use coupled with temperature and rainfall variations over the decades are the main drivers promoting the mongoose expansion. The geographic barriers and the human infrastructures functioned as barriers for mongoose expansion and have shaped its distribution. The expansion of the Egyptian mongoose across the Portuguese territory was due to a variety of factors. Our results suggest a rapid shift in species range in response to land-use and climate changes, underlining the close link between species ranges and a changing environment.

## Introduction

Knowledge on the mechanisms controlling species distribution patterns is central in ecology [1,2,3]. For assessing those patterns, one can use different methods, including ecological surveys, museum specimens, bibliographical records, and interviews [e.g. 4–7]. By unravelling such patterns and identifying range shifts, both contractions and expansions, it is possible to create accurate measures for conservation and management with the emphasis on the species-environment relationship [8]. The diversity of factors affecting such distributional patterns have been categorized as either biotic—e.g. competition, predation, parasitism [e.g. 9,10,11]-, and abiotic e.g.—climatic, topographic, land-use [e.g. 12–17]-, and they are linked to time-space dynamics [18]. Animal and plant species have specific ecological requirements for their survival, and literature shows that the same factors impact different species at different levels, depending on temporal and spatial scales [e.g. 19].

More specifically, several studies assert human presence, anthropogenic activities and urban infrastructures as some of the major causes of declines and contractions in wildlife populations due to their consequences for habitat fragmentation [20,21,22]. However, other authors present a more optimistic scenario, indicating that the conservation of wild species might be possible even where human presence is strong [23,24].

Human-mediated changes to land-cover are now ubiquitous across the globe and are drastically transforming landscapes and, consequently, altering species distributions [e.g. 25,26,27]. Many studies have dealt with the impact of land use changes on species distribution in Mediterranean Europe [e.g. 28–32].

In recent decades, climate change has also been proven as a major cause of shifts in species’ ranges [e.g. 33–36]. There is now ample evidence that climate change may lead to range contractions in many organisms [37], either by reducing their optimal climatic envelope or due to the encroachment of the optimal climatic envelope of better-adapted invaders [38].

Under a scenario of anthropogenic-driven environmental changes, is essential to understand the processes beneath range shifts. Also, for expanding species, there may be a need to control for their potential impacts on the newly occupied territory [39–42]. Shifts in species ranges may be modelled according to stochastic processes but also according to deterministic mechanisms, such as those resulting from a response to changes in the environment [43]. Most studies in the literature focused on understanding rapid range expansions of exotic species during processes of invasion [44,45,46]. However, it is equally important to understand how and why a native species that was confined for a long time within a specific range, suddenly expands into new areas [e.g. 47,48]. This is the case of the Egyptian mongoose, *Herpestes ichneumon* (Linnaeus, 1758). Though traditionally considered an exotic herpestid species in the Iberian Peninsula that was intentionally introduced by the invading Moors in the Middle Ages [49,50,51], recent genetic studies showed that this carnivore probably naturally settled in Iberia during the Late Pleistocene sea-level fluctuations [52].

In Africa, the species is widely distributed, albeit absent from the Sahara Desert, the wet forests of central and West Africa and the deserts of South Africa [53,54]. It is also present in the Middle East, including Syria, Jordan, the Palestinian Territories, and Israel [55].

Whether of Pleistocene origin or a Moorish introduction, until recently the Egyptian mongoose was restricted to the south of the Tagus River, which probably acted as a natural barrier to the expansion into northern territories [56]. However, in the late 1980s the Egyptian mongoose rapidly started expanded northwards beyond the Tagus River in Portugal [57,58], considerably extending the limits of its traditionally known range in this country [59].

Knowing that species ranges are limited by several factors, including vegetation cover and climatic aspects, and that those may change over time and space, we hypothesize that the sudden expansion of the Egyptian mongoose in Portugal is due to changes in either the i) barrier effects of human infrastructure and topographic features; ii) availability of suitable areas due to climate change; iii) availability of suitable areas and expansion corridors due to changes in land use; or iv) a combination of all of the above.

Using field data, questionnaires and literature, we analysed the patterns of the species’ expansion over the last three decades (1980–1990; 1990–2000; 2000–2010) and evaluated the relationship between the explanatory variables chosen for each hypothesis and the expansion of the Egyptian mongoose to newly colonised areas.

The Egyptian mongoose is known to favour the Mediterranean *maquis* and it is well-adapted to the climate of southern Portugal, which presents warmer temperatures in comparison to central and northern regions. Hence, we trust that any occurred alterations across the Portuguese range especially comprising vegetation cover and climatic aspects consequently affected the distribution of this species. The thorough analysis of the species expansion across the last three decades will largely contribute with new information related with the ecology of the Egyptian mongoose, by emphasizing the link between the changing environment and the occurrence of this species.

## Methods

### Study area

The study area encompasses the entire Portuguese continental territory (35°57’- 42°10’ N, 6°12’- 9°29’ E). Central and northern areas are characterised by a mountainous landscape with the highest altitude in the Iberian Central Mountain Chain at Serra da Estrela (1993 m). Southern areas are characterised by flatlands and two major mountain chains: Serra de S. Mamede (1027 m) and Serra de Monchique (902 m). Climate and vegetation vary with the biogeographic sub-region: the Atlantic Mid-European Sub-region in the northwest has a temperate and humid climate, wet summers and high levels of precipitation. In the Atlantic Mid-European Sub-region the dominant species are oaks (*Quercus* sp.), beeches (*Fagus* spp.), birches (*Betula* spp.), ashes (*Fraxinus* spp.) and maples (*Acer* spp.). The remaining and the majority of the territory is within the Western Mediterranean sub-region, with hot and dry summers and with high precipitation levels in other seasons, ranging from 350 mm to 1500 mm. The Mediterranean Sub-region is characterised by oaks, mastic (*Pistacia lentiscus*), laurustinus (*Virbunum tinus*), olive trees (*Olea europaea*), carob trees (*Ceratonia siliqua*) and *Phillyrea angustifolia* [60]. However, in recent decades, intensive monocultures of eucalyptus (*Eucalyptus globulus*) and maritime pine (*Pinus pinaster*) have been planted throughout the country, significantly modifying forest composition [61].

### Data collection, analysis and modelling procedures

#### Presence-absence data

We added new data on the presence-absence (P/A) of the Egyptian mongoose to that collected and published in Barros [57] and Barros and Fonseca [58]. The new presence-absence data was assessed by collected specimens from hunting activities. For more details concerning the P/A data see Figs A, B and C in S1 Fig. We also used additional information on the distribution of the mongoose available in the literature for confirmation purposes [56,59,62].

We used those P/A data to calculate the occupied area from one decade to another and we generated three maps to reflect the distribution of the Egyptian mongoose in the following periods: between 1980 and 1990, between 1990 and 2000 and between 2000 and 2010. Since the presence of the Egyptian mongoose in Portugal was collected according to municipality, all P/A data was projected in a map with the Portuguese territory divided by this administrative unit. We then evaluated the expansion between the units where the mongoose was present in the previous period to the new units occupied by the mongoose in the following one. Maps were built in ArcGIS Version 10.2.

#### Data variables

We collected a total of 17 variables based on their possible effect on mongoose expansion in each studied period: Weighted Urban Area per unit (WUrban), Weighted Roads Extension per unit (WRoad), Weighted Human Population Density per unit (PopDens), Mean Altitude (MeanAltit), Maximum Altitude (MaxAltit), Minimum Altitude (MinAltit), Mean Slope (MeanSlope), Maximum Slope (MaxSlope), Land Ruggedness (TRness), Weighted River Extension per unit (WRiver), Temperature variation for each decade (ΔT), Rainfall variation for each decade (ΔR), Weighted Open Areas per unit (WOpenArea), Weighted Closed Areas per unit (WClosedArea), Weighted Forest Areas per unit (WForest), Weighted Scrub Areas per unit (WScrub) and Weighted Crop Areas per unit (WCrop). All variables were selected based on previous studies describing habitat requirements that influence mongoose presence and also on species having similarly-described habitat requirements [63–66]. We did not include prey availability as a variable in our study due to the generalist nature of the diet of this species, including small mammals, reptiles, amphibians, invertebrates and occasionally berries and other fruits, and its significant variation across its distribution range [67,68]. The Egyptian mongoose is considered an opportunistic species, as it usually preys on the most abundant items available, which also causes significant variation in its diet along the year [69].

Data from all the variables was obtained for each studied period. We retrieved human population density for each period from the Instituto Nacional de Estatística website (www.ine.pt/); data on road extension for each period was assessed from the Instituto Geográfico Português website (www.igeo.pt); geographic data was downloaded from the U.S. Geological Survey website (http://srtm.usgs.gov/index.php); river extension was assessed via Sistema de Informação Nacional de Recursos Hídricos (http://snirh.apambiente.pt/); climatic data for each period was compiled from the European Climate Assessment and Dataset (ECAD) website (http://eca.knmi.nl/); and land cover and vegetation variables for each period were retrieved from the Corine Land Cover, with a spatial resolution (pixel width) of 250 m (http://www.eea.europa.eu/publications/COR0-landcover).

#### Analysis and modelling procedures

To reduce the risk of overfitting [70], an exploratory analysis was made by calculating the Spearman rank correlation to rank the variables in each studied temporal range. Variables showing a correlation above 0.7 were eliminated. We then used hierarchical partitioning analysis (HPA) [71,72], also for each temporal range, to select uncorrelated potential explanatory variables from those described above. HPA separates up to twelve variables with high independent correlations with the dependent variable from variables that show a high pairwise correlation with the dependent variable but that is due to the joint action of other independent variables [72]. This analysis was done using the R package ‘hier.part’ [73]. Models with more than nine explanatory variables, as in our study, may present a “minor rounding error” [73]. In identical cases like ours, some incongruities were found in terms of the ranking of the independent and co-dependent contributions of the variables depending on the order they enter the hierarchical analysis [74]. To correct these errors, Olea et al. [74] suggest that models should be run at least 100 times whilst reordering the variables. We adopted this approach in our study and then ranked the variables according to the number of times they showed the highest independent contribution towards the variation of the response variable, i.e. Egyptian mongoose expansion across the three periods. Once we were able to select a subset of significant potential predictors, we then grouped the variables in different groups based on each one of the explanatory hypotheses. We considered different hypotheses aiming to evaluate the partitioning of the variance in relation to the response variable. Four hypothesis were considered: one hypothesis gathering variables related with anthropogenic activities and natural barriers (WUrban, WRoad and MeanAltit), one gathering climatic variables (ΔT and ΔR), one grouping environmental variables related with land use and their alteration across the three decades (WForest, WScrub, WCrop, WOpenArea and WClosedArea), and a Global Hypothesis, this final hypothesis being a combination of all the variables from the other three hypotheses (Table 1). We then used generalized linear models (GLM) with a binomial error distribution and logit link function to evaluate which hypothesis and set of variables best modelled the expansion of the Egyptian mongoose in each period. GLM is a rather flexible and robust technique, least susceptible to over-fitting than other methods (e.g. classification trees, regression splines) [75]. Also, the GLM approach is able to deal with response variables that are not normally distributed [76].

**Table 1:** Potential explanatory variables and their corresponding values for every hypothesis with their influence on Egyptian mongoose expansion in each temporal range.

				Temporal range		
Hypothesis (*abbreviation*)		Explanatory variables (*abbreviation*)		1980–1990	1990–2000	2000–2010
			Unit	Mean		
Global (*Global*)	Anthropogenic and Geographic Barriers (*AnthrGeo*)	Weighted Urban Area per unit (*WUrban*)	m^2^	0.0	0.1	0.0
		Weighted Extension of Roads per unit (*WRoad*)	m	*WRoad1*	*WRoad2*	*WRoad3*
				0.1	0.5	0.6
		Mean Altitude (MeanAltit)	m	109.9	202.2	416.1
	Climate Change (*Clim*)	Temperature variation (*ΔT*)	°C	*ΔT90_80*	*ΔT00_90*	*ΔT10_00*
				0.4	3.6	-3.1
		Rainfall variation (*ΔR*)	mm	*ΔR90_80*	*ΔR00_90*	*ΔR10_00*
				11	-13.3	10.4
	Land Use Changes (*LUChanges*)	Weighted Forest Area per unit (*WForest*)	m^2^	0.3	0.2	0.3
		Weighted Scrub Area per unit (*WScrub*)	m^2^	0.2	0.3	0.4
		Weighted Crop Area per unit (*WCrop*)	m^2^	0.4	0.4	0.3
		Weighted Open Area per unit (*WOpenArea*)	m^2^	0.5	0.5	0.5
		Weighted Closed Area per unit (*WClosedArea*)	m^2^	0.3	0.3	0.5

With the aim of truly reflecting the expansion of the species from one decade to another, P/A data for every period was arranged in separated matrices. We selected P/A data for each period solely reflecting the newly colonised areas in each period. Thus, for the 1980–1990 matrix, P/A data reflects the presence of the species in the areas occupied in the first period, plus the absence of the species in areas occupied in the following two periods; for the 1990–2000 matrix, presence data corresponds to the colonisation of new areas in 1990–2000 and the absence of the species from those same areas in 1980–1990; and finally, for the 2000–2010 matrix, we selected presence data for the species in the newly-occupied areas in that period, plus the absence of the species from those same areas in the previous periods. Therefore, P/A data in each period does not reflect the cumulative distribution of the species across each decade, but the incremental expansion of the species that reflects the newly-occupied areas from one decade to another. Every model was run for these incremental areas against the variables associated with those increments. We used the Akaike Information Criterion (AIC) to rank the best-fitted models. All analyses were done in R Version 3.1.2 [77].

## Results

### Egyptian mongoose in Portugal

**Fig 1:**
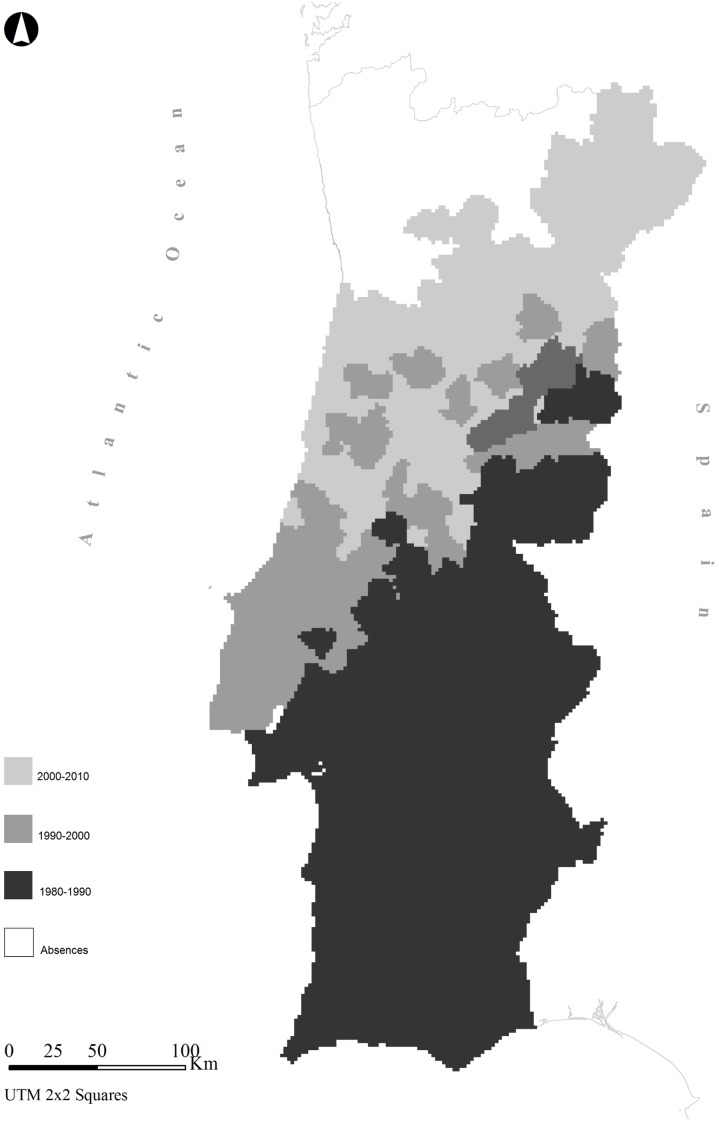
Map representing the expansion of the Egyptian mongoose across 1980–1990, 1990–2000 and 2000–2010 periods.

**Fig 2:**
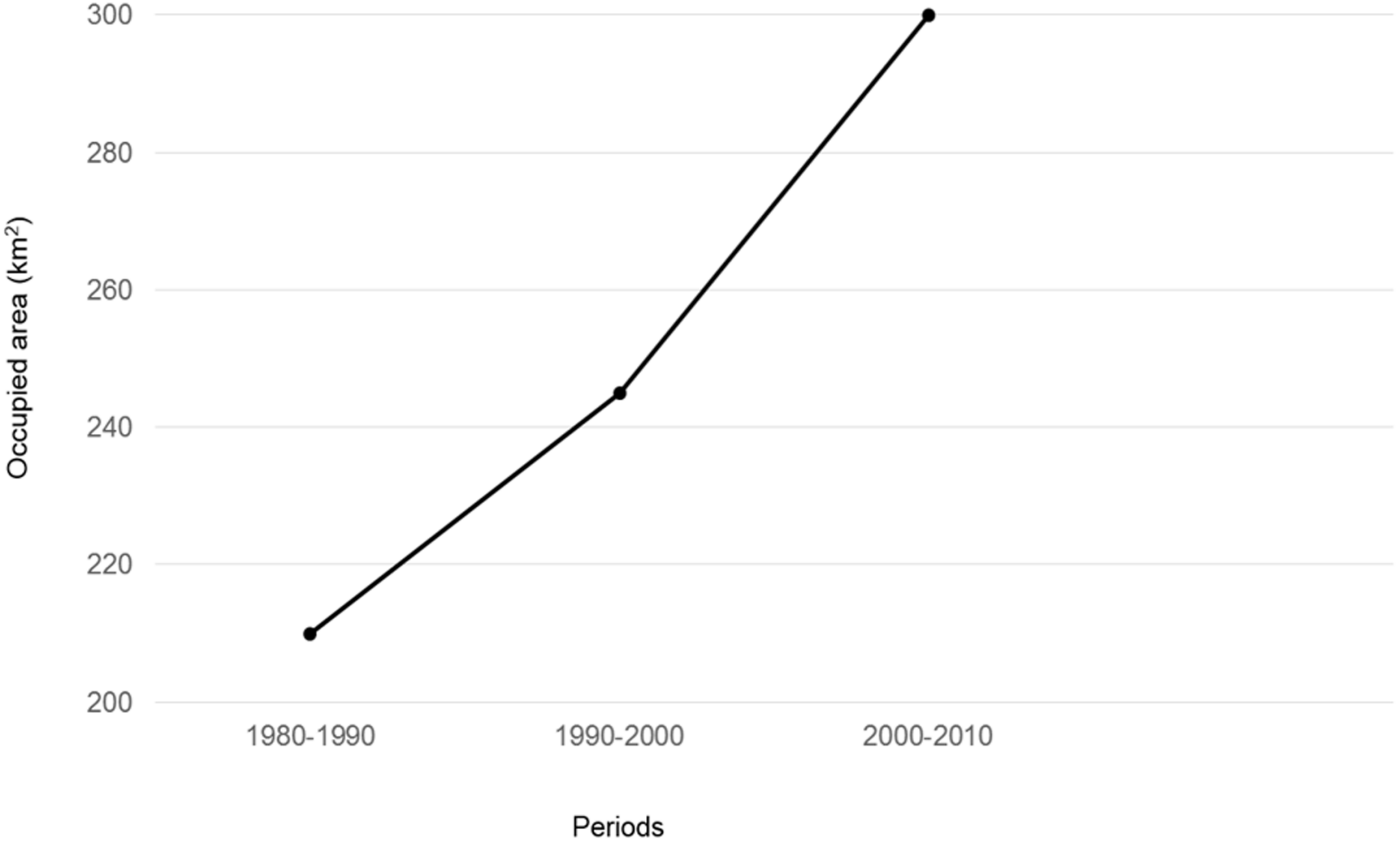
Graphical representation of the occupied area by the Egyptian mongoose in 1980–1990, 1990–2000 and 2000–2010 periods.

The species expanded more intensively towards the central [both inland and along the coast) and north-eastern territories (Fig 1). From the 1980s to the 1990s, the range of the Egyptian mongoose in Portugal increased from approximately 210 km^2^ to 245 km^2^ (Fig 2). The steepest increase in the range of the species was documented in the last decade—between 2000 and 2010—when its expansion continued further northeast, but also towards coastal areas with an increase of 55 km^2^ relative to 1990–2000. Currently, the area occupied by the Egyptian mongoose in Portugal is ca. 300 km^2^, and includes almost the entire Portuguese territory, with the exception of the northwest tip of the country.

### Factors behind the sudden expansion

**Fig 3:**
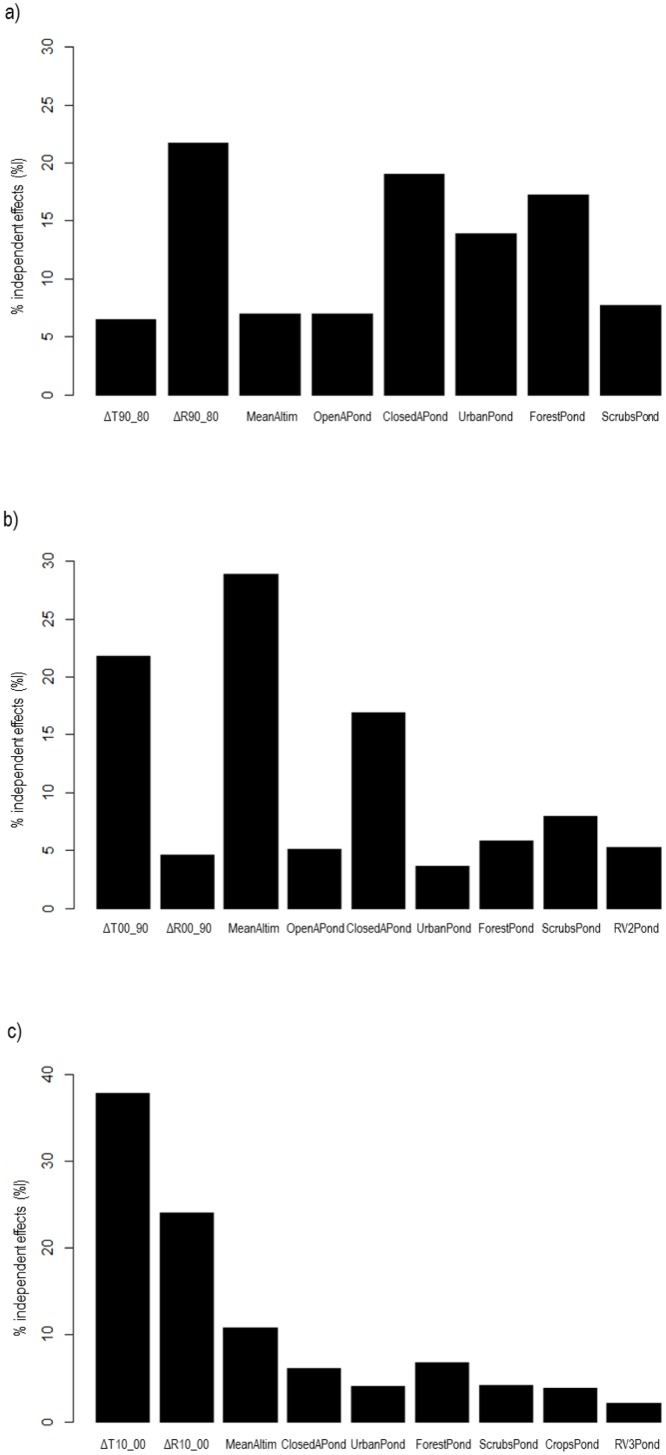
Graphics representing the percentage of the independent effect of the studied variables analyzed in (a) 1980–1990, (b) 1990–2000 and (c) 2000–2010 periods, assessed by hierarchical partition analysis (HPA).

Highly correlated variables were eliminated. We eliminated MaxAltit, MinAltit, MeanSlope, MaxSlope, TRness and PopDens in the three temporal ranges. WCrop was eliminated from the 1980–1990 and 1990–2000 ranges, as well as RV1Pond from 1980–1990 ranges. The results of the HPA showed that for the 1980–1990 range the variables contributing most to the variation of the response variable were WClosedArea, ΔR90_80, WForest, WUrban, WScrub, MeanAlti, ΔT90_80 and WOpenArea. For the 1990–2000 range, the highest-contributing variables were MeanAltit, ΔT00_90, WClosedArea, WScrub, WForest, WRoad2, ΔR00_90 and WUrban. For 2000–2010, ΔT10_00, ΔR10_00, MeanAltit, WForest, WClosedArea, WScrub, WCrop, WUrban and WRoad3 contributed most to variation (Fig 3). The variable WRiver was removed from every analyzed temporal range and WOpenArea was removed from the 2000–2010 range because their independent contributions to variation of the response variable were lower to the models’ goodness-of-fit.

By testing the three hypotheses separately (AnthrGeo; Clim; LUChanges), in tandem (AnthrGeo_Clim; AnthrGeo_LUChanges; Clim_LUChanges) and combined (Global) (Table 1), GLM analyses generated a total of 57 models for the analyzed temporal ranges (see S1 Table). Table 2 shows the best models for each temporal range. The best models for the three temporal ranges include variables from the three hypotheses (Global Model), with the majority of the variables highly correlated with the expansion of the Egyptian mongoose. We found WUrban, WForest, WScrub, WOpenArea, WClosedArea and ΔR90_80 as highly correlated variables with the expansion of the species between 1980 and 1990 (p > 0.000). MeanAltit was the least correlated variable (p > 0.05) in this temporal range. MeanAltit, ΔT00_90, WClosedArea and WScrub were found to be highly correlated for 1990–2000 (p > 0.000), while WForest and WOpenArea (p > 0.001), WUrban (p > 0.01), ΔR00_90 (p > 0.05) and WRoad2 (p > 0.1) were less correlated. In the temporal range between 2000 and 2010, MeanAltit, WClosedArea, WForest. WScrub and ΔT10_00 were highly correlated with the expansion of the species (p > 0.000), whilst WCrop was less correlated (p > 0.001).

**Table 2:** Best model generated by GLM for each studied period.

Period	Estimate	Std. Error	z value	Pr(>|z|)
*1980–1990*				
(Intercept)	10.590	3.014	3.514	0.000 ***
MeanAlt	-0.003	0.001	-1.882	0.060 .
WUrban	-15.187	3.525	-4.308	1.64e-05 ***
WForest	15.028	3.663	4.103	4.07e-05 ***
WScrub	9.341	2.384	3.918	8.91e-05 ***
WOpenArea	-12.136	3.388	-3.582	0.000 ***
WClosedArea	-22.723	4.005	-5.673	1.40e-08 ***
ΔR90_80	-0.126	0.034	-3.690	0.000 ***
*1990–2000*				
(Intercept)	6.670	2.652	2.518	0.012 *
MeanAltit	-0.007	0.001	-5.913	3.35e-09 ***
WUrban	-6.401	3.055	-2.095	0.036 *
WRoad2	-1.550	0.989	-1.568	0.116967
ΔT00_90	0.452	0.124	3.638	0.000 ***
ΔR00_90	-0.040	0.020	-1.950	0.051146 .
WOpenArea	-8.407	2.881	-2.918	0.004 **
ClosedAPon	-14.307	3.021	-4.735	2.19e-06 ***
WForest	6.473	2.497	2.592	0.009 **
WScrub	6.487	1.877	3.456	0.000 ***
*2000–2010*				
(Intercept)	-23.295	0.699	-3.332	0.000 ***
MeanAltit	-0.004	0.001	-5.195	2.04e-07 ***
WClosedArea	-73.690	15.658	-4.706	2.52e-06 ***
WCrop	28.106	0.998	2.817	0.005 **
WForest	108.074	19.718	5.481	4.23e-08 ***
WScrub	89.356	16.289	5.486	4.12e-08 ***
ΔT10_00	-0.443	0.048	-9.180	< 2e-16 ***

significance codes:

0 ‘***’

0.001 ‘**’

0.01 ‘*’

0.05 ‘.’

0.1 ‘ ’

## Discussion

There is a clear link between the physical environment and the distribution of a species, in which the influencing factors may assume a major or minor role depending on a geographic-time gradient [78,79]. This explains why the best models found for each temporal range included variables expressing different effects on mongoose expansion in the Portuguese territory. Variables explaining mongoose expansion were mutable over time, except MeanAltit. We found variables with the same effect across the Portuguese range in the 1980–1990 and 1990–2000 temporal ranges [MeanAltit (-), WForest (+), WScrub (+), and WClosedArea (-). WUrban (-), WOpenArea (-) and ΔR (-)]. Temperature (ΔT) showed a variable effect in the 1990–2000 and 2000–2010 periods. WRoad2 (-) and WCrop (+) were only significant in the 1990–2000 and 2000–2010 periods, respectively. Our results suggest that the expansion of the Egyptian mongoose in the Portuguese territory is mostly associated with anthropogenically-driven changes in the landscape.

### Human infrastructure vs mongoose expansion

In Portugal, rural areas exhibit lower human densities compared to the coastal region, where urbanisation is more intense [80]. This dichotomy started to be even more evident in the 1990s with migration from inland to the coast, resulting in significant rural depopulation [81,82]. We found a highly significant negative effect of urban areas on mongoose expansion across the two first studied temporal ranges (Table 2). Similarly to the majority of wild carnivores [83,84], in the Iberian Peninsula the Egyptian mongoose avoids anthropic-disturbed areas with high human population densities [56]. In the first decade, the species was mainly present in the south-east where it was absent from intensely urbanized areas. In the second decade, the expansion was most notable towards the coast, and particularly the Lisbon district, but it was still absent from highly populated areas.

We also found a negative correlation between road density and the expansion of the species during the 1990–2000 period. Road networks can negatively affect wildlife and ecosystems [85–88], limiting animal movements and causing a significant number of deaths by road- kills [89–93]. In the specific case of the Egyptian mongoose, data shows that this species is frequently reported as road-kill across the species’ distributional range [94; unpublished data]. This underlines the negative impact the increasing construction of roads in the second period must have had on the Egyptian mongoose populations. In fact, as a result of European Union policies, there was a considerable investment in public works associated with the construction of highways and other main roads in order to facilitate access and transportation across the Portuguese territory during the 1990s (European Commission 2014 - http://ec.europa.eu/legislation/index_en.htm). In the 1980–1990 period, roads covered a significantly lower area of the species distributional range than in other decades (see Table 1), re-enforcing the idea that road construction had a significant negative effect on the expansion of the mongoose in the following periods.

### The hurdle effect of altitude

During the 1980s the mongoose was confined to southern areas, with a tendency to occupy rural territories (see Fig 1). Low altitude plateaus characterise the majority of these territories, where mountainous landscapes are scarce. However, greater altitudes are found in central and northern areas, which were occupied by the mongoose during 1990–2000 and 2000–2010. Besides the altitude variation across the Portuguese range, we found a negative correlation between altitude and the expansion of the Egyptian mongoose across the three studied periods. Climatic factors show large spatial discrepancies in mountainous areas and affect habitat conditions [95,96], leading to changes in floral composition [97,98,99] and distressing animal species richness and abundance [100–103]. Indeed, the Egyptian mongoose seems to avoid high altitudes [56], and seems to prefer the Mediterranean *maquis* [65], where conditions of shelter, food availability and climatic elements are ideal for the species.

### The lands they are a-changing

Land-use has been changing in the Mediterranean in the last four decades [104,105,106], and in the case of the Iberian Peninsula these changes have been highly significant. Mediterranean *maquis* is commonly present in the southern territories of Portugal and it is a well-known and common habitat with an essential role for several medium-sized carnivores [e.g. 63,107,108].

Although Mediterranean woodland can also be found in central and north-eastern areas of Portugal, the establishment of monocultures of *Eucalyptus* sp. and *Pinus* sp. began to be commonplace throughout Portugal in the last two decades and is rapidly replacing the Mediterranean *maquis* [61,109]. Mongoose presence is commonly linked to the Mediterranean landscape across the Iberian Peninsula, and an important part of its activities [e.g. foraging, resting and sheltering) are displayed in areas with *maquis* vegetation [53,65,66,110,111], which explains the positive correlation between forest areas and mongoose expansion in the three decades. Besides the crescent implementation of monocultures of pine and eucalyptus, we believe these land-use changes has also benefitted the Egyptian mongoose, as studies confirm that the Egyptian mongoose is found in areas with both tree species [57,65]. This suggestion could explain the positive correlation between mongoose expansion in the last two study periods and forest areas.

Moreover, shrub areas became more frequent in the last three decades due to rural depopulation of the Portuguese countryside [112]. This trend was accentuated when national policies were influenced by the Common Agricultural Policy, which focused on productivity and greatly transformed traditional farming schemes (European Commission 2014 - http://ec.europa.eu/legislation/index_en.htm). In fact, during the 1980s this led to significant land abandonment, especially evident in the interior of the country, resulting in a decrease in human population density, while simultaneously promoting recovery of non-cultivated vegetation. Higher densities of Mediterranean scrublands began to be more frequent across the Portuguese countryside and this led to an increase in the availability of sheltering and foraging resources for the Egyptian mongoose in areas where they were traditionally less available, promoting the expansion of the species. There was an additional decrease in the area used for agriculture due to increasing land prices in the coast and the north-east [113]. Decreased crop production also promoted the expansion of shrub areas and the appearance of more heterogeneous and patchy mosaics, which are important for medium-sized carnivores like the Egyptian mongoose [e.g. 114–117].

We found a negative correlation between open areas and the species’ expansion in the three periods. This result was predictable due to the diurnal habits of the species in the Iberian Peninsula and its avoidance of open areas, which can expose it to human interference and natural predators [65,110,111]. However, we found the same effect between closed areas and mongoose expansion. We hypothesize that this simultaneous negative correlation with open and closed areas is due to the categorization of Corine Land Cover data. If we defined ‘Closed Areas’ by a single category grouping forest areas and ‘Open Areas’ as shrub areas, crops and agricultural areas, we would discard other variables that we did not find them to be preponderant for answering the main questions of our study.

### The hotter and drier, the better

The Egyptian mongoose is present in the Mediterranean and in the Afrotropic Region [53]. An extensive range of temperatures and precipitation occur in these regions. Yet, in Africa—from where the Egyptian mongoose radiated [52]-, the species is absent from the Sahara desert [54], where the climate is characterised by extreme temperatures and long dry seasons [118,119]. The Egyptian mongoose is also absent from central-African rainforests [54], characterised by the highest rainfall levels in that continent [82]. It seems then that this species avoids extreme environments in both its ancient and present distributional range.

We found a positive correlation between temperature and mongoose expansion in Portugal during the 1990–2000 period, leading to the assumption that the expansion was driven also by temperature variations. Climate conditions in southern areas traditionally occupied by the mongoose are characterised by the highest annual temperatures [120]. Furthermore, several studies have already recorded climate change as an impact factor on species ranges in Portugal [121,122]. Because the Egyptian mongoose has a preference for these warmer climates, this seems to explain why the species was confined to southern Portugal for several decades; however, during 1990–2000, a striking temperature increase of 3.6°C was registered in central territories (see Table 1). Species undergoing expansion have greater chances of survival in areas where climatic conditions are analogous to those in their native ranges [123], so this temperature shift seems to have had a role in the sudden expansion of the mongoose by transforming the northern territories into more climatically suitable areas for this species.

Still, a negative correlation between temperature and mongoose presence was found during the 2000–2010 temporal range (Table 2). Temperature is often linked to altitude, and the territories occupied in that temporal range are frequently colder as they are greatly characterised by a mountainous landscape. A temperature decrease of -3.1°C was recorded in the colonised territories during 2000–2010. We believe that the expansion of the Egyptian mongoose was negatively affected by temperature in the last period, as the species is not adapted to colder climates.

Similarly to temperature, variation in rainfall limits the presence of many species in a wide variety of habitats [e.g. 124]. We found rainfall to be a limiting factor for species expansion in the 1980–1990 and 1990–2000 temporal ranges (Table 2), as the Egyptian mongoose is better adapted to dry conditions. Southern areas have lower precipitation levels compared with northern areas [121], but in the first studied decade, we found that the Egyptian mongoose was limited by rainfall and maintained its range in areas where rainfall levels were more favourable. Between 1990 and 2000, rainfall levels decreased significantly (see Table 1), providing a drier environment for the species, but still had a limiting effect on mongoose dispersal.

The current distribution of the Egyptian mongoose overlaps what is presently recognized as Mediterranean sub-region, while the species is still absent from the northwest, included in the Atlantic sub-region. These regions have distinct climates, independently of the climatic variations found in the three temporal ranges studied here. Our data clearly indicates that the Mediterranean climate is much more suitable for the Egyptian mongoose.

## Conclusion

The expansion of the Egyptian mongoose over the three last decades was influenced by a multitude of factors. We found that climatic factors, human-mediated factors, geographic and habitat features played a critical role in the sudden expansion and current distribution of this carnivore in the Portugal. A similar pattern of interactions between several factors impacting on a species’ distribution has been observed for other carnivores in Europe. Barbosa et al. [20] found spatial, environmental and human-mediated factors were considerable influences on the distribution of the otter (*Lutra lutra*); Virgós et al. [125] revealed that altitude, climatic conditions and land cover play an important role for the presence of the genet (*Genetta genetta*); and Zabala et al. [126] found that the expansion of the European mink (*Mustela lutreola*) was driven by environmental, land-use and interspecific competition.

Our study had some limitations. We compared three different spatial contexts but this cannot be overcome in studies concerning species range expansions across a temporal scale. Also, we restricted our study to Portugal and did not proceed with the analysis of the entire Iberian Peninsula due to the absence of updated data concerning the current distribution of the Egyptian mongoose in Spain. The expansion of the species in Spain might also be occurring, nevertheless, the inclusion of incomplete data of the current situation of the species in the Spanish territory would weaken our study.

Nevertheless, we believe our study contributes with valuable information to understanding the drivers underlying the distributional pattern of the Egyptian mongoose in Portugal. The applications of this study should reflect a top-down approach, from contributing to management guidelines for worldwide expanding species in an altered environment, to a finer scale, where the creation of potential management guidelines for the expanding mongoose populations in Portugal should be considered.

## Supporting Information

S1 Fig Presence/Absence data analysed in each studied period for each municipality. Each map represents the combinations of analysed data for each municipality. Figure A) Presence/Absence data for 1980–1990 period; Figure B) Presence/Absence data for 1990–2000 period; Figure C) Presence/Absence data for 2000–2010 period. Data concerning inquiries, direct observations, and museum records/stuffed animals was collected by Barros [57] and Barros and Fonseca [58]; Hunting records were obtained from Instituto da Conservação da Natureza e das Florestas (ICNF) and were collected by Barros [57] and Barros and Fonseca [58]; Data concerning animal collection were obtained from collected mongooses from hunting activities in the last decade, under the project ‘Genetic assessment of a successful invasion: Population genetics of the Egyptian mongoose (*Herpestes ichneumon*) in Portugal (TDC/BIA-BEC/104401/2008) (see details in http://www.cesam.ua.pt/index.php?menu=200&language=eng&tabela=projectosdetail&projectid=380); References were used for additional information on the distribution of the species across the three decades [56,59,62]. Absence data across the three decades were also confirmed by inquiries [57–58], hunting records [57–58] and bibliographic references [56,59,62].
(DOC)

S1 Table Models generated for each studied temporal range and each AIC and ΔAIC value. Bold italic models indicate the selected model for each period.
(DOC)
