# Supplementary material for: Following The Trail: Factors Underlying the Sudden Expansion of the Egyptian Mongoose (Herpestes ichneumon) in Portugal
Source: PLoS One. 2015 Aug 12;10(8):e0133768. doi: 10.1371/journal.pone.0133768 (PMC4534455; doi:10.1371/journal.pone.0133768)
Supplement: S1 Fig — Each map represents the combinations of analysed data for each municipality. Figure A) Presence/Absence data for 1980–1990 period; Figure B) Presence/Absence data for 1990–2000 period; Figure C) Presence/Absence data for 2000–2010 period. Data concerning inquiries, direct observations, and museum records/stuffed animals was collected by Barros [57] and Barros and Fonseca [58]; Hunting records were obtained from Instituto da Conservação da Natureza e das Florestas (ICNF) and were collected by Barros [57] and Barros and Fonseca [58]; Data concerning animal collection were obtained from collected mongooses from hunting activities in the last decade, under the project ‘Genetic assessment of a successful invasion: Population genetics of the Egyptian mongoose (Herpestes ichneumon) in Portugal (TDC/BIA-BEC/104401/2008) (see details in http://www.cesam.ua.pt/index.php?menu=200&language=eng&tabela=projectosdetail&projectid=380); References were used for additional information on the distribution of the species across the three decades [56,59,62]. Absence data across the three decades were also confirmed by inquiries [57–58], hunting records [57–58] and bibliographic references [56,59,62]. (DOC) [file pone.0133768.s001.doc]

**S1 File. Presence/Absence data analysed in each studied period for each municipality.** Each map represents the combinations of analysed data for each municipality. Figure A) Presence/Absence data for 1980-1990 period; Figure B) Presence/Absence data for 1990-2000 period; Figure C) Presence/Absence data for 2000-2010 period. Data concerning inquiries, direct observations, and museum records/stuffed animals was collected by Barros [57] and Barros and Fonseca [58]; Hunting records were obtained from Instituto da Conservação da Natureza e das Florestas (ICNF) and were collected by Barros [57] and Barros and Fonseca [58]; Data concerning animal collection were obtained from collected mongooses from hunting activities in the last decade, under the project ‘Genetic assessment of a successful invasion: Population genetics of the Egyptian mongoose (*Herpestes ichneumon*) in Portugal (TDC/BIA-BEC/104401/2008) (see details in <http://www.cesam.ua.pt/index.php?menu=200&language=eng&tabela=projectosdetail&projectid=380>); References were used for additional information on the distribution of the species across the three decades [56,59,62]. Absence data across the three decades were also confirmed by inquiries [57-58], hunting records [57-58] and bibliographic references [56,59,62].


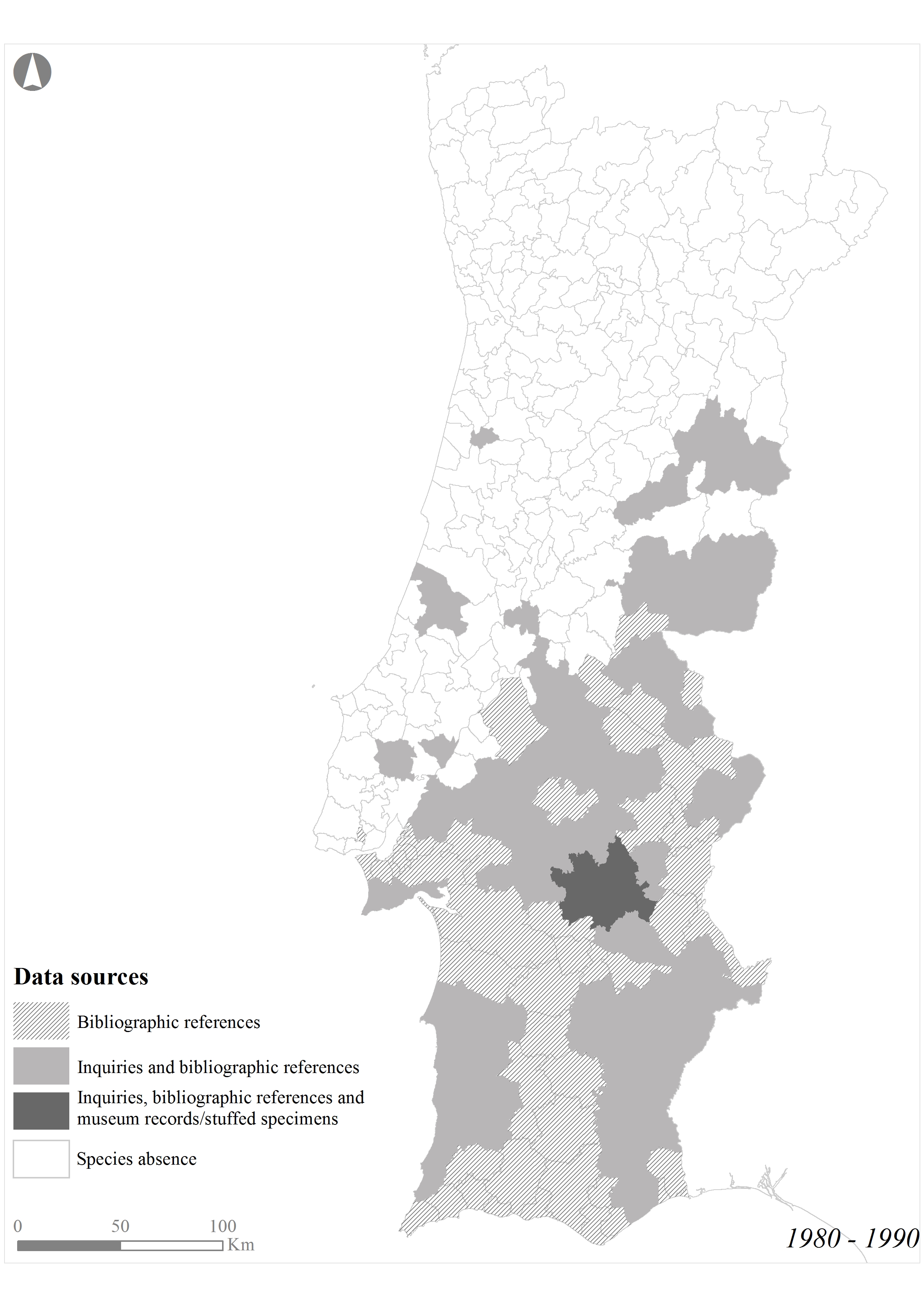


a)


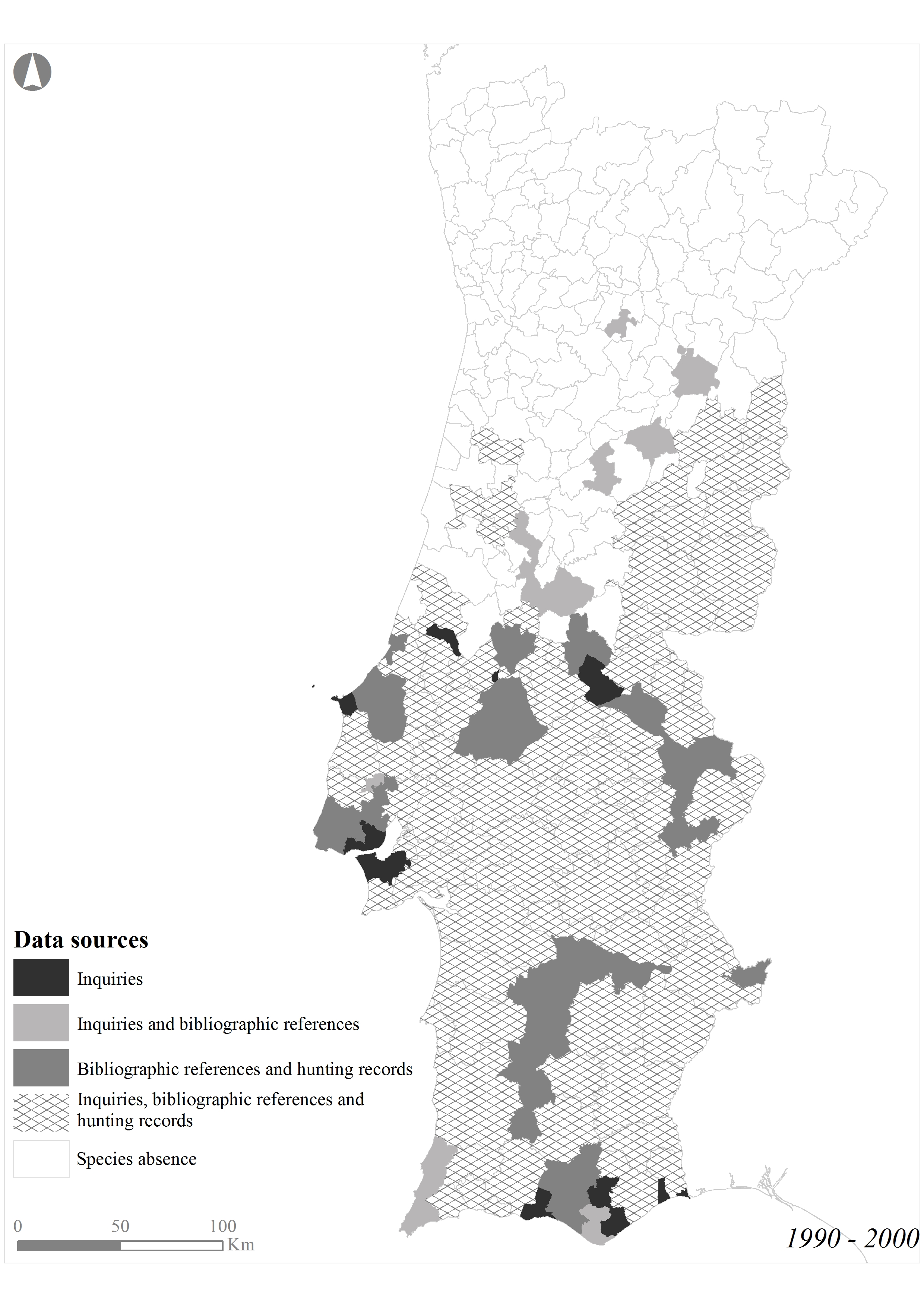


b)


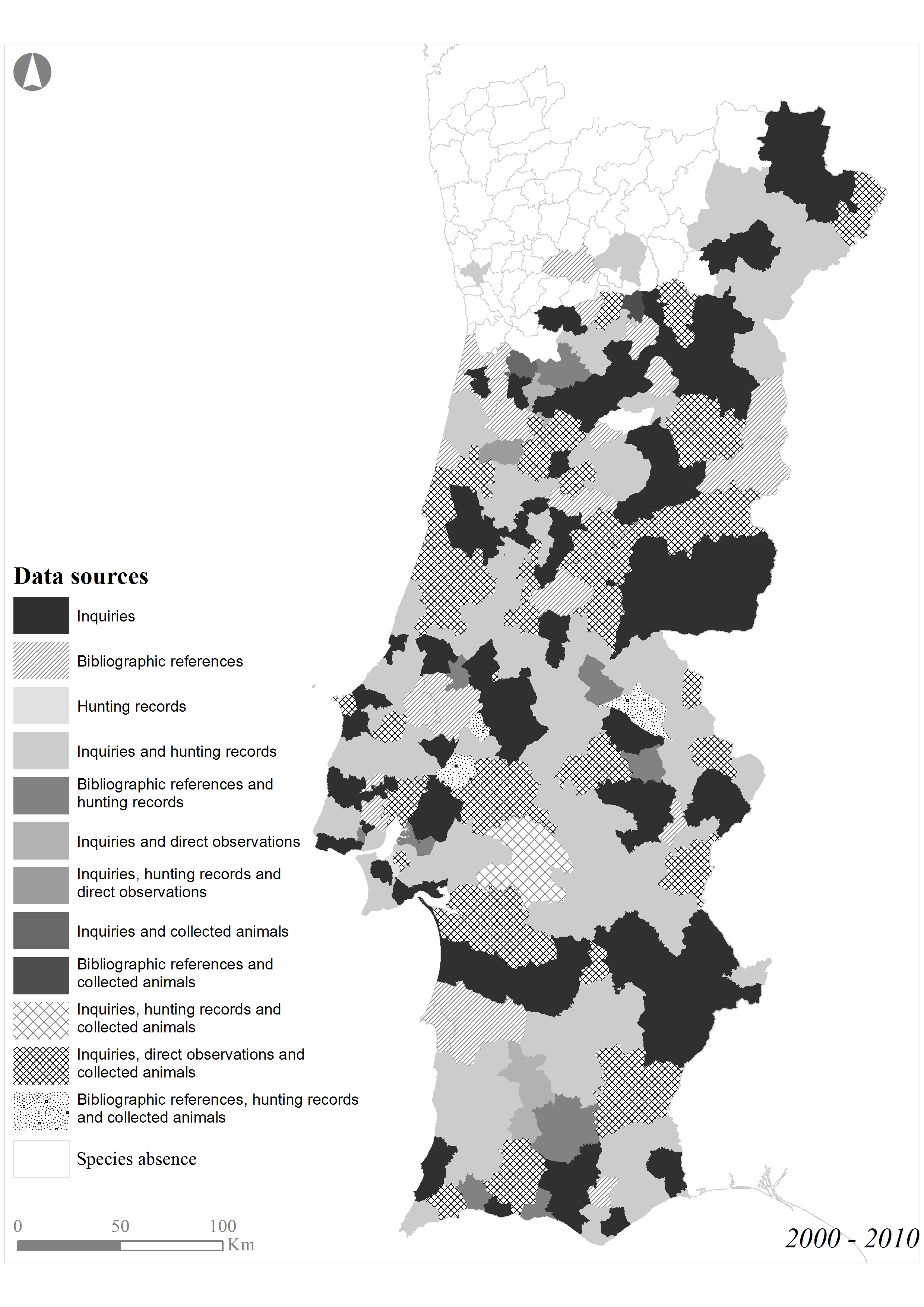


c)
