# Supplementary material for: Following The Trail: Factors Underlying the Sudden Expansion of the Egyptian Mongoose (Herpestes ichneumon) in Portugal
Source: PLoS One. 2015 Aug 12;10(8):e0133768. doi: 10.1371/journal.pone.0133768 (PMC4534455; doi:10.1371/journal.pone.0133768)
Supplement: S1 Table — Bold italic models indicate the selected model for each period. (DOC) [file pone.0133768.s002.doc]

**S1 Table.** Models generated for each studied temporal range and each AIC and ΔAIC value (the table continues in the next page). Bold italic models indicate the selected model for each period.

| **Model code** | **Variables** | **AIC** | **ΔAIC** |
| --- | --- | --- | --- |
| **80_90** | | | |
| MAnthrGeo_80_90_a | MeanAltit+WUrban | 297.95 | 108.79 |
| MClim_80_90_a | ΔT90_80+ΔR90_80 | 289.50 | 100.34 |
| MClim_80_90_b | ΔR90_80 | 288.56 | 99.40 |
| MLUChanges_80_90_a | WOpenArea+ClosedPond+WForest+WScrub | 250.38 | 61.22 |
| MLUChanges_80_90_b | ClosedPond+WForest+WScrub | 251.06 | 234.06 |
| MAnthrGeoClim_80_90_a | MeanAltit+WUrban+ΔT90_80+ΔR90_80 | 249.96 | 60.80 |
| MAnthrGeoClim_80_90_b | MeanAltit+WUrban+ΔR90_80 | 248.07 | 58.91 |
| MAnthrGeoClim_80_90_c | WUrban+ΔR90_80 | 251.75 | 62.59 |
| MAnthrGeoLUChanges__80_90_a | MeanAltit+WUrban+WForest+WScrub+WOpenArea+WClosedAread | 203.39 | 14.23 |
| MAnthrGeoLUChanges__80_90_b | WUrban+WForest+WScrub+WOpenArea+WClosedAread | 221.71 | 32.55 |
| MClimLUChanges_80_90_a | ΔT90_80+ΔR90_80+WForest+WScrub+WOpenArea+WClosedAread | 214.52 | 25.36 |
| MClimLUChanges_80_90_b | ΔT90_80+ΔR90_80+WForest+WScrub+WClosedAread | 212.55 | 23.39 |
| MClimLUChanges_80_90_c | ΔR90_80+WForest+WScrub+WClosedAread | 214.08 | 24.92 |
| MGlobal_80_90a | MeanAltit+WUrban+WForest+WScrub+WOpenArea+WClosedArea+ΔT90_80+ΔR90_80 | 189.70 | 0.54 |
| ***MGlobal_80_90b*** | ***MeanAltit+WUrban+WForest+WScrub+WOpenArea+WClosedArea+ΔR90_80*** | ***189.16*** | ***0*** |
| MGlobal_80_90c | WUrban+WForest+WScrub+WOpenArea+WClosedArea+ΔR90_80 | 190.97 | 1.81 |
| **90_00** | | | |
| MAnthrGeo_90_00_a | MeanAltit+WUrban+Road2Pond | 403.40 | 88.54 |
| MAnthrGeo_90_00_b | MeanAltit+Road2Pond | 401.90 | 87.04 |
| MClim_90_00_a | ΔT00_90+ΔR00_90 | 417.66 | 102.80 |
| MClim_90_00_b | ΔT00_90 | 419.51 | 104.65 |
| MLUChanges_90_00_a | WOpenArea+WClosedArea+WForest+WScrub | 395.71 | 80.85 |
| MLUChanges_90_00_b | WClosedArea+WForest+WScrub | 395.39 | 80.53 |
| MLUChanges_90_00_c | WClosedArea+WForest | 399.22 | 84.36 |
| MAnthrGeoClim_90_00_a | MeanAltit+WUrban+Road2Pond+ΔT00_90+ΔR00_90 | 341.67 | 26.81 |
| MAnthrGeoClim_90_00_b | MeanAltit+WUrban+Road2Pond+ΔT00_90 | 344.37 | 29.51 |
| MAnthrGeoClim_90_00_c | MeanAltit+Road2Pond+ΔT00_90 | 344.89 | 30.03 |
| MAnthrGeoClim_90_00_d | MeanAltit+ΔT00_90 | 352 | 37.14 |
| MAnthrGeoLUChanges__90_00_a | MeanAltit+WUrban+Road2Pond+WOpenArea+WClosedArea+WForest+WScrub | 351.78 | 36.92 |
| MAnthrGeoLUChanges__90_00_b | MeanAltit+WUrban+WOpenArea+WClosedArea+WForest+WScrub | 349.88 | 35.02 |
| MClimLUChanges_90_00_a | ΔT00_90+ΔR00_90+WForest+WScrub+WOpenArea+WClosedArea | 371.61 | 56.75 |
| MClimLUChanges_90_00_b | ΔT00_90+WForest+WScrub+WOpenArea+WClosedArea | 369.98 | 55.12 |
| MClimLUChanges_90_00_c | ΔT00_90+WForest+WOpenArea+WClosedArea | 370.23 | 55.37 |
| MClimLUChanges_90_00_d | ΔT00_90+WForest+WClosedArea | 375.03 | 60.17 |
| ***MGlobal_90_00a*** | ***MeanAltit+WUrban+Road2Pond+ΔT00_90+ΔR00_90+WOpenArea+WClosedArea+WForest+WScrub*** | ***314.86*** | ***0*** |
| MGlobal_90_00b | MeanAltit+WUrban+ΔT00_90+ΔR00_90+WOpenArea+WClosedArea+WForest+WScrub | 315.32 | 0.46 |
| MGlobal_90_00c | MeanAltit+WUrban+ΔT00_90+WOpenArea+WClosedArea+WForest+WScrub | 315.47 | 0.61 |
| MGlobal_90_00d | MeanAltit+WUrban+ΔT00_90+WOpenArea+WClosedArea+WScrub | 321.37 | 6.51 |
| **2000_2010** | | | |
| MAnthrGeo__00_10_a | MeanAltit+WUrban+Road3Pond | 535.20 | 125.15 |
| MClim_00_10_a | ΔT10_00+ΔR10_00 | 474.07 | 64.02 |
| MClim_00_10_b | ΔT10_00 | 472.47 | 62.42 |
| MLUChanges_00_10_a | WScrub+WForest+WCrop+WClosedArea | 544.77 | 134.72 |
| MLUChanges_00_10_b | WScrub+WForest+WClosedArea | 551.28 | 141.23 |
| MAnthrGeoClim_00_10_a | MeanAltit+WUrban+Road3Pond+ΔT10_00+ΔR10_00 | 439.28 | 29.23 |
| MAnthrGeoClim_00_10_b | MeanAltit+WUrban+ΔT10_00+ΔR10_00 | 437.94 | 27.89 |
| MAnthrGeoClim_00_10_c | MeanAltit+WUrban+ΔT10_00 | 437.32 | 27.27 |
| MAnthrGeoClim_00_10_d | MeanAltit+ΔT10_00 | 452.29 | 42.24 |
| MAnthrGeoLUChanges__00_10_a | MeanAltit+WUrban+Road3Pond+WClosedArea+WCrop+WForest+WScrub | 504.83 | 94.78 |
| MAnthrGeoLUChanges__00_10_b | MeanAltit+WUrban+Road3Pond+WClosedArea+WForest+WScrub | 504.60 | 94.55 |
| MAnthrGeoLUChanges__00_10_c | MeanAltit+WUrban+Road3Pond+WClosedArea+WForest | 519.90 | 109.85 |
| MClimLUChanges_00_10_a | ΔT10_00+ΔR10_00+WForest+WScrub+WCrop+WClosedArea | 441.22 | 31.17 |
| MClimLUChanges_00_10_b | ΔT10_00+WForest+WScrub+WCrop+WClosedArea | 439.97 | 29.92 |
| MClimLUChanges_00_10_c | ΔT10_00+WForest+WScrub+WClosedArea | 444.53 | 34.48 |
| MGlobal_00_10a | MeanAltit+WUrban+Road3Pond+WClosedArea+WCrop+WForest+WScrub+ΔT10_00+ΔR10_00 | 414.99 | 4.94 |
| MGlobal_00_10b | MeanAltit+WUrban+WClosedArea+WCrop+WForest+WScrub+ΔT10_00+ΔR10_00 | 413.04 | 2.99 |
| MGlobal_00_10c | MeanAltit+WClosedArea+WCrop+WForest+WScrub+ΔT10_00+ΔR10_00 | 411.10 | 1.05 |
| ***MGlobal_00_10d*** | ***MeanAltit+WClosedArea+WCrop+WForest+WScrub+ΔT10_00*** | ***410.05*** | ***0*** |
| MGlobal_00_10e | MeanAltit+WClosedArea+WForest+WScrub+ΔT10_00 | 416.61 | 6.56 |
